# Methotrexate-cytarabine-dexamethasone combination chemotherapy with or without rituximab in patients with primary central nervous system lymphoma

**DOI:** 10.18632/oncotarget.17101

**Published:** 2017-04-13

**Authors:** Xuefei Sun, Jing Liu, Yaming Wang, Xueyan Bai, Yuedan Chen, Jun Qian, Hong Zhu, Fusheng Liu, Xiaoguang Qiu, Shengjun Sun, Nan Ji, Yuanbo Liu

**Affiliations:** ^1^ Department of Hematology, Beijing Tiantan Hospital, Capital Medical University, Beijing, China; ^2^ Department of Neurosurgery, Navy General Hospital, Beijing, China; ^3^ Beijing Neurosurgical Institute, Beijing Tiantan Hospital, Capital Medical University, Beijing, China; ^4^ Department of Radiation Therapy, Beijing Tiantan Hospital, Capital Medical University, Beijing, China; ^5^ Neuroimaging Center, Beijing Tiantan Hospital, Capital Medical University, Beijing, China; ^6^ Department of Neurosurgery, Beijing Tiantan Hospital, Capital Medical University, Beijing, China

**Keywords:** primary central nervous system lymphoma, rituximab, high-dose methotrexate, cytarabine, survival

## Abstract

**Purpose:**

High-dose methotrexate based chemotherapy is the standard treatment for patients with newly diagnosed primary central nervous system lymphoma (PCNSL). The role of rituximab is controversial because of its large size, which limits its penetration of the blood-brain barrier. In this study, we investigated the efficacy and tolerability of adding rituximab to methotrexate-cytarabine-dexamethasone combination therapy (RMAD regimen).

**Results:**

The patients treated with RMAD had a complete remission rate of 66.7% after induction chemotherapy; this rate was only 33.3% in patients treated with MAD alone (*p* = .011). The most common grade 1–3 adverse events were similar and included hematologic toxicity, increased aminotransferase levels, and gastrointestinal reactions. Multivariate analysis revealed that rituximab treatment was associated with longer progression-free survival (PFS, *p* = .005) but not overall survival (OS). Additionally, we observed that elevated serum lactate dehydrogenase was associated with shorter OS and PFS.

**Materials and Methods:**

We retrospectively analyzed 60 immunocompetent patients with newly diagnosed PCNSL at Beijing Tiantan Hospital, Capital Medical University from January 2010 to June 2016. Twenty-four patients received 3–6 courses of 3.5 g/m^2^ methotrexate on day 1; 0.5–1 g/m^2^ cytarabine on day 2; and 5–10 mg dexamethasone on days 1, 2 and 3. Thirty-six patients received the same combination plus rituximab 375 mg/m^2^ on day 0. All patients repeated the treatment every 3 weeks.

**Conclusions:**

High-dose methotrexate based chemotherapy with rituximab yields a higher complete remission rate and does not increase serious toxicities. PFS benefits from the addition of rituximab. OS has an increasing trend in patients treated with rituximab without statistical significance.

## INTRODUCTION

Primary central nervous system lymphoma (PCNSL) is an aggressive non-Hodgkin lymphoma located in the brain, leptomeninges, spinal cord, cerebrospinal fluid (CSF) and intraocular structures [1, 2]. The tumor is usually diffuse large B cell lymphoma (DLBCL) and is associated with worse prognosis than systemic lymphomas of the same type. Although high-dose methotrexate (HD-MTX) is the most effective drug for PCNSL, usually with recommended dose of 3.5 g/m^2^ every 2–3 weeks, the median overall survival (OS) is 10–20 months, and progression-free survival (PFS) is 12–13 months [3–6]. Thus, the ability of the addition of other drugs to HD-MTX to improve the outcome of HD-MTX treatment has been examined [7–9].

Cytarabine (Ara-C) kills proliferating cells in the S-phase of the cell cycle. The administration of HD-Ara-C after HD-MTX (MA) increases cytotoxicity. A randomized phase II trial comparing HD-MTX alone with HD-MTX combined with HD-Ara-C showed that the addition of Ara-C increased the response rate and extended OS [10]. Rituximab is an anti-CD20 hybrid monoclonal antibody that is active against various types of B-cell lymphoma. The addition of rituximab to the cyclophosphamide, doxorubicin, vincristine and prednisone (CHOP) regimen has become a cornerstone of therapy for systemic DLBCL [11]. However, there are many concerns regarding the ability of this antibody to cross the blood-brain barrier (BBB). Preliminary evidence has demonstrated that R-CHOP may not significantly prevent central nervous system (CNS) dissemination of systemic DLBCL compared with CHOP alone [12–14]. However, in CNS lymphoma patients, the use of intravenous rituximab can induce responses in contrast-enhancement lesions, likely in lesions in which there is substantial disruption of the BBB [15]. The precise role of rituximab in PCNSL remains controversial [16–19] and has not been defined.

In this study, we retrospectively analyzed the characteristics of 60 patients with newly diagnosed PCNSL and evaluated the role of adding rituximab to the methotrexate-cytarabine-dexamethasone (MAD) regimen as a first-line chemotherapy for PCNSL.

## RESULTS

### Patient characteristics and treatment

The characteristics of the PCNSL patients are described in Table 1. The diagnosis was obtained by stereotactic biopsy (81.7%), surgery (15.0%), or CSF (3.3%). All PCNSLs were proven to be DLBCL. The male-female ratio was 1.5:1 for the 60 patients. The median patient age was 57 years (range 11 to 83 years, 27 were ≥ 60 and 33 were < 60 years old). Ten patients (16.7%) had an Eastern Cooperative Oncology Group (ECOG) performance status of 0 or 1. The remaining 50 patients (83.3%) had an ECOG performance status of 2–4. Multiple brain lesions were observed in 35 patients (58.3%), and deep brain structures were compromised in 50 patients (83.3%). Serum lactate dehydrogenase (LDH) levels were elevated in 20 (33.3%) of the 60 patients. The patients were treated according to their will and financial conditions. In total, 24 patients received MAD induction chemotherapy, and 36 received the RMAD regimen. A total of 25 (41.7%) patients received consolidation treatment. Eighteen (30.0%) patients received WBRT as salvage therapy, and 17 (28.3%) did not pursue further treatment.

**Table 1:** Clinical characteristics of PCNSL patients

		Total(*n* = 60, %)	RMAD(*n* = 36, 60%)	MAD(*n* = 24, 40%)	*P* value
Age	≥ 60	27 (45.0)	17 (47.2)	10 (41.7)	0.672
	< 60	33 (55.0)	19 (52.8)	14 (58.3)	
Gender	Male	36 (60.0)	22 (61.1)	14 (58.3)	0.830
	Female	24 (40.0)	14 (38.9)	10 (41.7)	
LDH	Elevated	20 (33.3)	11 (30.6)	8 (33.3)	0.821
	Normal	40 (66.7)	25 (69.4)	16 (66.7)	
Number of lesions	1	26 (43.3)	15 (41.7)	11 (45.8)	0.750
	At least 2	34 (56.7)	21 (58.3)	13 (54.2)	
ECOG performance status	0 to 1	10 (16.7)	6 (16.7)	4 (16.7)	1.000
	At least 2	50 (83.3)	30 (83.3)	20 (83.3)	
Deep structure involvement	Presence	50 (83.3)	32 (88.9)	18 (75.0)	0.178
	Absence	10 (16.7)	4 (11.1)	6 (25.0)	
Diagnosis	Stereotactic biopsy	49 (81.7)	30 (83.3)	19 (79.2)	0.321
	Surgery	9 (15.0)	4 (11.1)	5 (20.8)	
	CSF	2 (3.3)	2 (5.6)	0 (0)	
Induction treatment response	CR	32 (53.3)	24 (66.7)	8 (33.3)	0.011
	Without CR	28 (46.7)	12 (33.3)	16 (66.7)	
Further treatment	Consolidation	25 (41.7)	17 (47.2)	8 (33.3)	0.267
	Salvage	18 (30.0)	8 (22.2)	10 (41.7)	
	not proceed	17 ((28.3)	11 (30.6)	6 (25.0)	
Pathology	DLBCL	60 (100.0)	36 (100.0)	24 (100.0)	0.992
	GCB	5 (8.3)	3 (8.3)	2 (8.3)	
	ABC	42 (70.0)	25 (69.4)	17 (70.8)	
	Unclassified	13 (21.7)	8 (22.2)	5 (20.8)	

Abbreviations: LDH, lactate dehydrogenase; ECOG, Eastern Cooperative Oncology Group; DLBCL, diffuse large B-cell lymphoma; GCB, germinal center B-cell-like; ABC, activated B-cell-like; RMAD, rituximab-methotrexate-cytarabine-dexamethasone; MAD, methotrexate-cytarabine-dexamethasone.

### Response and survival

All 60 patients received at least 3 cycles of induction chemotherapy, and 46 patients completed 6 cycles. The response rates for chemotherapy are shown in Table 2. A total of 32 patients achieved CR (53.3%) after 3 cycles of induction chemotherapy. There were also 11 PR (18.3%), 11 SD (18.3%), and 6 PD (10%) patients. The 24 patients treated with MAD had the following outcomes: 8 CR (33.3%), 9 PR (37.5%), 4 SD (16.7%), and 3 PD (12.5%). The 36 patients treated with RMAD had the following outcomes: 24 CR (66.7%), 2 PR (5.6%), 7 SD (19.4%), and 3 PD (8.3%). The patient characteristics (age, sex, ECOG, LDH, number of lesions, involvement of deep structures) did not differ between patients treated with RMAD and MAD, except for the response rate. The induction chemotherapy regimen was significantly associated with the CR rate (RMAD: 66.7% vs. MAD: 33.3%, *p* = .011) (Table 1).

**Table 2:** Response to induction chemotherapy

Response	All patients (*n* = 60)	Patients with RMAD (*n* = 36)	Patients with MAD (*n* = 24)
Complete remission	32 (53.3%)	24 (66.7%)	8 (33.3%)
Partial remission	11 (18.3%)	2 (5.6%)	9 (37.5%)
Stable disease	11 (18.3%)	7 (19.4%)	4 (16.7%)
Progression disease	6 (10.0%)	3 (8.3%)	3 (12.5%)
Died during therapy	0 (0)	0 (0)	0 (0)

**Figure 1:**
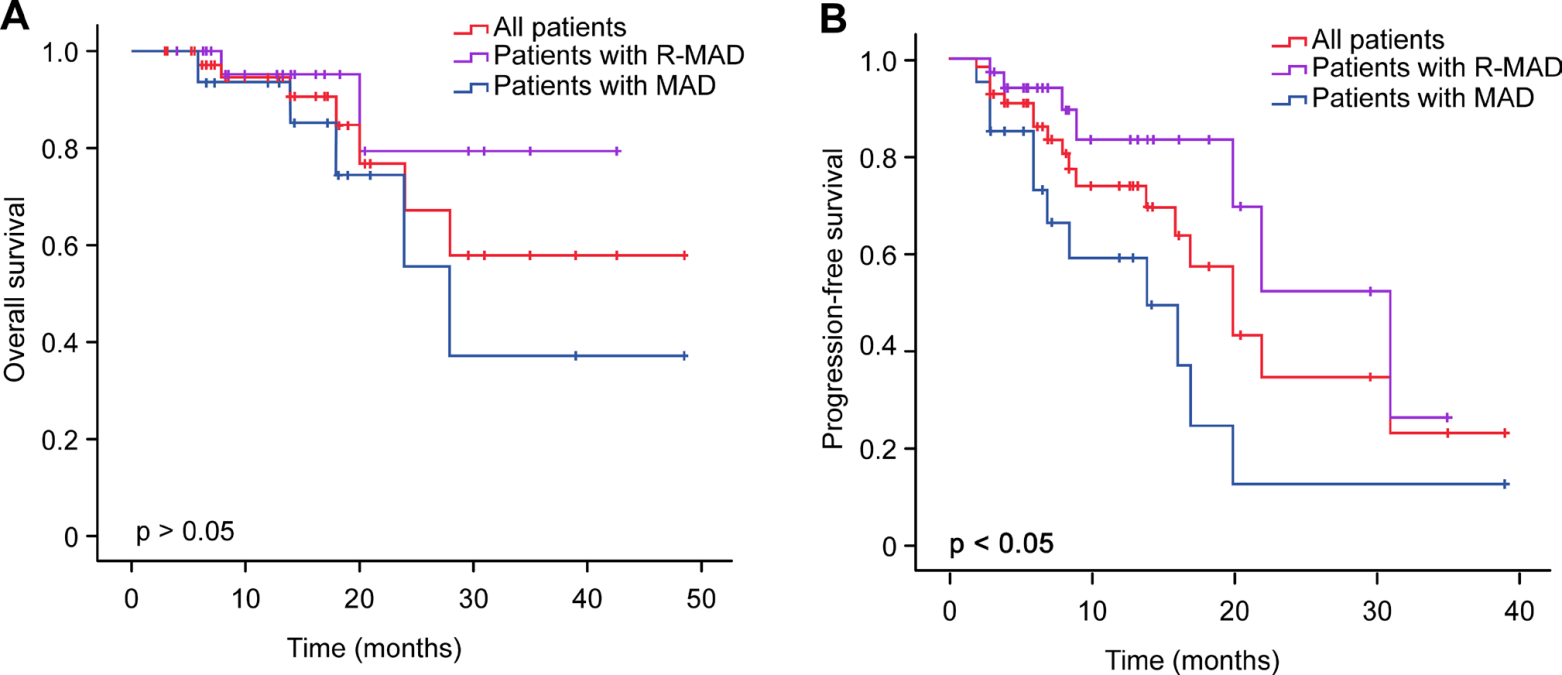
Kaplan-Meier analysis of OS and PFS in PCNSL patients and comparison of OS and PFS between groups with or without rituximab by the log-rank test

**Figure 2:**
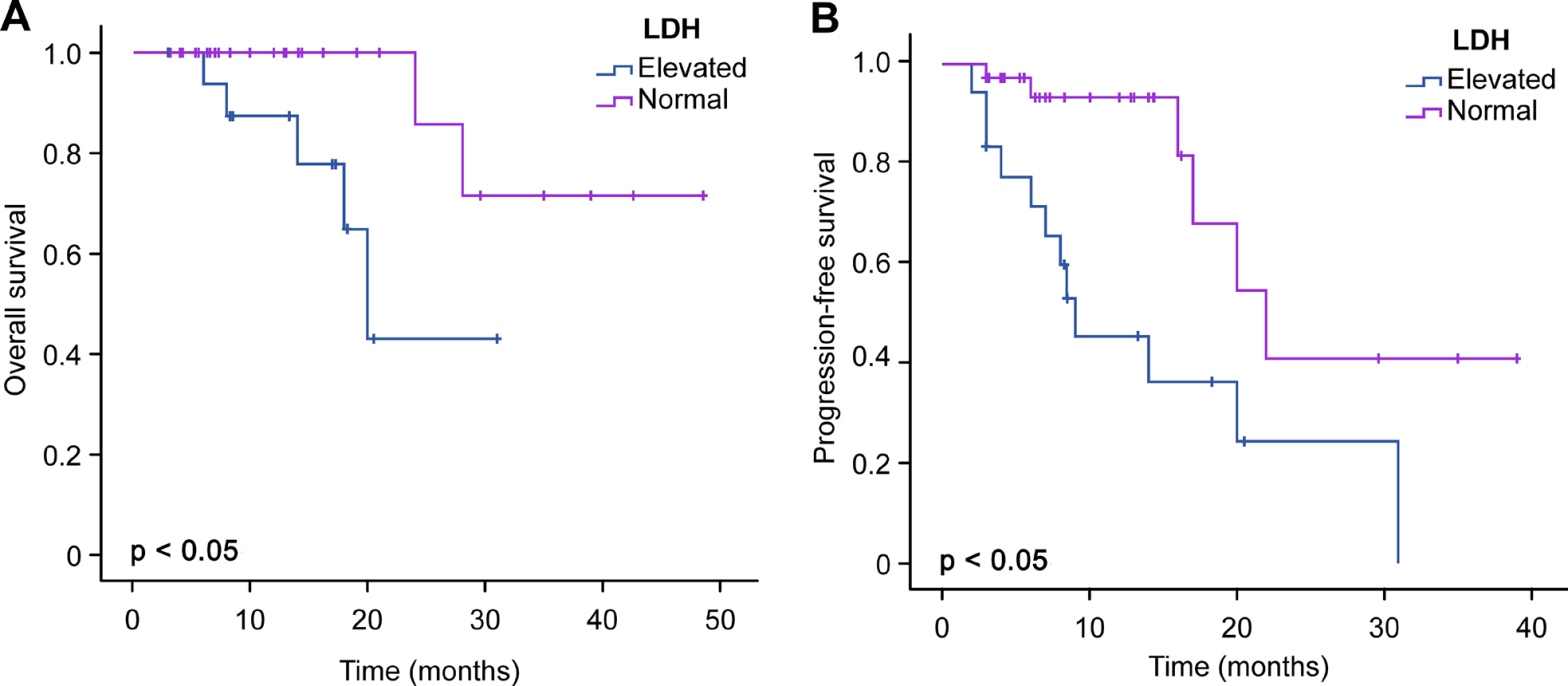
Comparison of OS and PFS between elevated LDH and normal LDH levels

Follow-up data were available for 54 patients. The 2-year PFS rate was 0.34, and the median PFS was 20.0 months (95% CI 15.22–24.78). The median OS for the 54 patients has not been reached. The estimated probability of OS at 4 years was 0.58 (range 0.31–0.85). The median OS in patients treated with RMAD has not been reached. However, in patients treated with MAD, the median OS was 28.0 months (95% CI 19.69–36.31). The median PFS in patients treated with RMAD was 31.0 months (95% CI 20.77–41.24). The median PFS in patients treated with MAD was 14.0 months (95% CI 4.93–23.07) (Table 3). Univariate analysis indicated that treatment with RMAD was associated with longer PFS (*p* = .015) but not OS (*p* = .176) (Figure 1). We also observed that elevated LDH was associated with shorter OS (*p* = .030) and PFS (*p* = .006) (Figure 2). Multivariate analysis revealed that the induction chemotherapy regimen and LDH level were independent risk factors for PFS. Multivariate analysis of OS identified only LDH as a prognostic indicator (Table 4).

**Table 3:** Overall survival and progression-free survival

	All patients (*n* = 54)	Patients with RMAD (*n* = 34)	Patients with MAD (*n* = 20)
Survival			
Median OS (months, 95% CI)	NR	NR	28.0 (19.69–36.31)
Median PFS (months, 95% CI)	20.0 (15.22–24.78)	31.0 (20.77–41.24)	14.0 (4.93–23.07)

Abbreviations: NR, not reached.

**Table 4:** Univariate and multivariate analyses of OS and PFS for patients (Cox test)

	OS						PFS					
Variable	Univariate analysis			Multivariate analysis			Univariate analysis			Multivariate analysis		
	HR	95% CI	*P*	HR	95% CI	*P*	HR	95% CI	*P*	HR	95% CI	*P*
Age	0.16	0.02–1.30	0.086	0.28	0.02–3.61	0.330	085	0.33–2.19	0.737	1.88	0.59–5.98	0.283
Gender	1.05	0.23–4.75	0.949	-	-	-	0.65	0.24–1.72	0.385	-	-	-
ECOG	0.39	0.04–3.54	0.404	0.72	0.03–19.03	0.843	2.12	0.28–16.03	0.469	2.27	0.28–18.41	0.444
LDH	0.15	0.03–0.83	0.030	0.06	0.00–0.97	0.048	0.25	0.09–0.66	0.006	0.10	0.03–0.37	0.001
Number of lesions	0.46	0.10–2.17	0.323	0.44	0.07–2.83	0.389	0.93	0.35–2.42	0.875	0.89	0.27–2.93	0.846
Deep structure involvement	2.65	0.27–25.94	0.403	4.99	0.16–160.88	0.365	1.07	0.24–4.79	0.932	1.11	0.22–5.63	0.897
Induction therapeutic regimen	3.12	0.60–16.19	0.176	10.42	0.83–131.27	0.070	3.25	1.25–8.44	0.015	5.23	1.64–16.70	0.005

Age, ≥ 60 y vs. < 60 y; Gender, male vs. female; ECOG, 0–1 vs. 2–4; LDH, elevated vs. normal; number of lesions, 1 vs. ≥ 2 lesions; deep structure involvement, presence vs. absence; induction therapeutic regimen, RMAD vs. MAD.

### Toxicity

The toxicities are summarized in Table 5. The most frequent toxicities after induction chemotherapy were hematologic toxicity (76.7%), elevated aminotransferase levels (48.3%), and gastrointestinal reactions (46.7%). The observed neurotoxicity was predominantly leukoencephalopathy (18.3%) and appeared in patients receiving at least 6 cycles of induction chemotherapy. There were no grade 4 toxicities or treatment-related deaths. The most common grade 1–3 adverse events were similar in both treatment groups. Patients treated with rituximab experienced more frequent anaphylaxis. There were 2 cases of skin allergy, 4 cases of fever, 1 case of respiratory tract allergy, and 1 case of interstitial pneumonitis among the 36 patients treated with rituximab.

**Table 5:** Toxicity graded according to the national cancer institute common toxicity criteria

Toxicity	All patients (*n* = 60)	Patients with RMAD (*n* = 36)	Patients with MAD (*n* = 24)
**Hematological toxicity**			
Neutropenia	42 (70.0%)	27 (75.0%)	15 (62.5%)
Infection	18 (30.0%)	10 (33.3)	8 (33.3%)
Anemia	7 (11.7%)	3 (8.3%)	4 (16.7%)
Thrombocytopenia	14 (23.3%)	9 (25.0%)	5 (20.8%)
**Liver toxicity**			
Aminotransferases elevated	29 (48.3%)	18 (50.0%)	11 (45.8%)
Bilirubin elevated	0	0	0
**Nephrotoxicity**			
Creatinine elevated	6 (10%)	4 (11.1%)	2 (8.3%)
Proteinuria	0	0	0
Hematuresis	0	0	0
**Gastrointestinal reaction**			
Mucositis/stomatitis	8 (13.3%)	5 (13.9%)	3 (12.5%)
Nausea	6 (10.0%)	3 (8.3%)	3 (12.5%)
Vomiting	1 (1.7%)	1 (2.8%)	0
Diarrhea	1 (1.7%)	1 (2.8%)	0
Constipation	9 (15.0%)	6 (16.7%)	3 (12.5%)
Inappetence	7 (11.7)	4 (11.1%)	3 (12.5%)
**Neurotoxicity**			
Leukoencephalopathy	11 (18.3%)	6 (16.7%)	5 (20.8%)
**Anaphylaxis**	7 (11.7%)	7 (19.4)	0
**Interstitial pneumonitis**	1	1 (2.8%)	0

## DISCUSSION

Rituximab is used in PCNSL patients due to its positive effect in non-CNS DLBCL. As a large protein, it poorly penetrates CNS. Following intravenous administration, the CSF levels of rituximab are approximately 0.1% of serum levels in patients with CNS lymphoma [20]. Although several studies have indicated that the addition of rituximab to MTX-based chemotherapy improves the survival of patients with PCNSL [16, 21, 22], the efficacy of rituximab in PCNSL need to be demonstrated further.

We retrospectively analyzed 60 PCNSL patients treated with RMAD or MAD in our single center. Comparing to MAD group, the high CR rate (67.7% vs 33.3%) and longer PFS (31 months vs 14 months) in RMAD group showed rituximab had active effect in PCNSL patients, consistent with prior analyses [18, 19, 23–27]. Comparisons of the median OS of patients treated with or without rituximab yielded different results: Madle et al. reported that rituximab treatment was an independent prognostic factor for OS in first-line treatment of PCNSL [28], whereas Mocikova et al. [18] and Kansara et al. [29] reported that the addition of rituximab to HD-MTX-based induction chemotherapy in PCNSL did not prolong median OS. Our result showed OS had an increasing trend in patients treated with rituximab, but the difference was not statistically significant (*p* = 0.176). The conflicting results need to be clarified by international randomized trials.

The backbone of chemotherapy regimen includes MTX and Ara-c. Previous studies have demonstrated that a reduced dose of systemic chemotherapy combined with rituximab can decrease the toxicity of systemic chemotherapy without altering efficacy [30–32]. In our study, we modified the chemotherapy regimens (MA) by reducing Ara-C to 0.5–1 g/m^2^ (1 dose), in contrast to the dose of 2 g/m^2^ (total of 4 doses) used by Ferreri et al. [10]. The result in RMAD group was a CR rate of 66.7% and no treatment-related mortalities, in contrast to the 46% CR rate and 8% treatment-related mortalities observed in patients treated with Ara-C 2 g/m^2^ (total of 4 doses). The result may benefit that we also delivered rituximab intravenously and used short-term treatment with small doses of dexamethasone. Meanwhile, the CR rate in MAD group was only 33.3% lower than Ferreri's result [10], it may be caused by the reduced Ara-c dose. Therefore, it is necessary to carry out prospective trial and stratified study to define the preferable dose of Ara-c.

The addition of rituximab to induction chemotherapy did not increase toxicity. The most common grade 1–3 adverse events were similar in the two groups and included neutropenia, anemia, thrombocytopenia, elevated aminotransferase levels, and gastrointestinal reactions. Rituximab is a biological agent and often causes allergic reactions, including skin allergy, fever, and respiratory tract allergy. Thus, dexamethasone and/or phenergan should be administered intravenously before administration of rituximab to prevent allergic reactions. In our study, one patient treated with rituximab developed interstitial pneumonitis. A chest CT revealed that the lesions resolved after administration of methylprednisolone 80 mg qd × 5d.

Our study has several limitations that have to be regarded. This study was limited by its small sample size and shorter follow-up time. The results of this present study was preliminary conclusions of single-center study, further prospective studies with cooperation of multi-center are necessary to confirm our results.

In conclusion, we compared the response rate to induction therapy, long-term outcomes, and toxicity between the two group of PCNSL patients with RMAD and MAD regimens to assess the role of rituximab. Our data indicated that adding rituximab to first-line induction chemotherapy can increase CR rate and significantly prolong PFS. OS showed an increasing trend in patients treated with rituximab, but the difference was not statistically significant. Therefore, this observation must be validated in prospective studies with a longer follow-up period.

## MATERIALS AND METHODS

### Patients

The clinical data of 60 immunocompetent patients with PCNSL from January 2010 to June 2016 were analyzed retrospectively. PCNSL was diagnosed by stereotactic biopsy, surgery or cerebrospinal fluid (CSF) analysis according to the Revised European-American Lymphoma and WHO classifications [33]. All patients in this study provided informed consent. This study was approved by the Beijing Tiantan Hospital Ethics Committee, Capital Medical University.

### Treatment

Induction chemotherapy consisted of HD-MTX, Ara-C, and dexamethasone (MAD). HD-MTX was administered intravenously at a dose of 3.5 g/m^2^ over 3 hours on day 1. Leucovorin rescue was initiated 24 hours after HD-MTX administration and administered every 6 hours until the methotrexate level was less than 0.10 μmol/L. Ara-C was administered intravenously at (0.5–1) g/m^2^ on day 2. The dose of Ara-C depended on the patient age and Eastern Cooperative Oncology Group (ECOG) performance status. Dexamethasone was administered at 5–10 mg on days 1, 2, and 3. The induction treatment consisted of 6 cycles of chemotherapy at 3-week intervals between cycles. Rituximab was administered at 375 mg/m^2^ on day 0 of every chemotherapy cycle.

The consolidation chemotherapy consisted of pemetrexed 900 mg/m^2^ administered on day 1 every 2 months for the first year and then every 6 months for year 2. Oral folic acid was administered at 400 μg daily 1 week before pemetrexed administration and continued for 3 weeks after the last dose. The patients also received intramuscular injections of 1000 μg vitamin B12 no less than 7 days before the administration of pemetrexed, and the injections were repeated every 9 weeks. The patients received two doses of 4 mg of oral dexamethasone daily for 3 days (day 0, day 1, and day 2).

Rescue WBRT was administered at a total dose of 45 Gy in 30 daily fractions of 1.5 Gy.

### Evaluation of response

The patient response was determined every 3 induction chemotherapy courses by contrast-enhanced Magnetic Resonance Imaging (MRI) of the brain. The responses were classified as complete remission (CR), partial remission (PR), stable disease (SD), and progression disease (PD), as described previously [30]. The patients who obtained CR after 6 cycles of induction chemotherapy received consolidation chemotherapy. The patients with PR, SD, PD, or relapse within one year received rescue WBRT. The patients with relapse after 1 year received the original induction chemotherapy. After completing induction therapy, the patients were evaluated by repeat contrast-enhanced MRI of the brain every 2 months for the first year and then every 4 months in years 2 and 3. The MRI was repeated every 6 months in years 4, 5 and 6.

OS was calculated from the date of diagnosis to the time of death from any cause. PFS was calculated from the start of treatment to the time of disease progression or death due to PCNSL.

### Evaluation of toxicity

The treatment toxicity was graded using the National Cancer Institute Common Toxicity Criteria version 3.0 [34].

### Statistics

The distribution of patient characteristics was evaluated using the chi-square test. The relationships between the induction chemotherapy regimen (MAD or RMAD) and clinicopathological variables were evaluated by Fisher's exact test and the χ^2^ test. Kaplan-Meier survival curves were obtained, and differences in OS or PFS were calculated using the log-rank test. The multivariate analysis for OS and PFS was conducted using Cox proportional hazards regression models. All statistical analyses were performed using the SPSS 17.0 software package, and *p* < 0.05 was considered statistically significant.

## References

[1] Batchelor T, Loeffler JS (2006). Primary CNS lymphoma. J Clin Oncol.

[2] Wang CC, Carnevale J, Rubenstein JL (2014). Progress in central nervous system lymphomas. Br J Haematol.

[3] Ekenel M, Deangelis LM (2007). Treatment of primary central nervous system lymphoma. Curr Treat Options Neurol.

[4] Jahnke K, Thiel E (2009). Treatment options for central nervous system lymphomas in immunocompetent patients. Expert Rev Neurother.

[5] Batchelor T, Carson K, O'Neill A, Grossman SA, Alavi J, New P, Hochberg F, Priet R (2003). Treatment of primary CNS lymphoma with methotrexate and deferred radiotherapy: a report of NABTT 96–07. J Clin Oncol.

[6] Herrlinger U, Schabet M, Brugger W, Kortmann RD, Kuker W, Deckert M, Engel C, Schmeck-Lindenau HJ, Mergenthaler HG, Krauseneck P, Benohr C, Meisner C, Wiestler OD (2002). German Cancer Society Neuro-Oncology Working Group NOA-03 multicenter trial of single-agent high-dose methotrexate for primary central nervous system lymphoma. Ann Neurol.

[7] DeAngelis LM, Yahalom J, Thaler HT, Kher U (1992). Combined modality therapy for primary CNS lymphoma. J Clin Oncol.

[8] O'Brien PC, Roos DE, Pratt G, Liew KH, Barton MB, Poulsen MG, Olver IN, Trotter GE (2006). Combined-modality therapy for primary central nervous system lymphoma: long-term data from a Phase II multicenter study (Trans-Tasman Radiation Oncology Group). Int J Radiat Oncol Biol Phys.

[9] DeAngelis LM, Seiferheld W, Schold SC, Fisher B, Schultz CJ (2002). Combination chemotherapy and radiotherapy for primary central nervous system lymphoma: Radiation Therapy Oncology Group Study 93–10. J Clin Oncol.

[10] Ferreri AJ, Reni M, Foppoli M, Martelli M, Pangalis GA, Frezzato M, Cabras MG, Fabbri A, Corazzelli G, Ilariucci F, Rossi G, Soffietti R, Stelitano C (2009). High-dose cytarabine plus high-dose methotrexate versus high-dose methotrexate alone in patients with primary CNS lymphoma: a randomised phase 2 trial. Lancet.

[11] Coiffier B, Lepage E, Briere J, Herbrecht R, Tilly H, Bouabdallah R, Morel P, Van Den Neste E, Salles G, Gaulard P, Reyes F, Lederlin P, Gisselbrecht C (2002). CHOP chemotherapy plus rituximab compared with CHOP alone in elderly patients with diffuse large-B-cell lymphoma. N Engl J Med.

[12] Feugier P, Virion JM, Tilly H, Haioun C, Marit G, Macro M, Bordessoule D, Recher C, Blanc M, Molina T, Lederlin P, Coiffier B (2004). Incidence and risk factors for central nervous system occurrence in elderly patients with diffuse large-B-cell lymphoma: influence of rituximab. Ann Oncol.

[13] Yamamoto W, Tomita N, Watanabe R, Hattori Y, Nakajima Y, Hyo R, Hashimoto C, Motomura S, Ishigatsubo Y (2010). Central nervous system involvement in diffuse large B-cell lymphoma. Eur J Haematol.

[14] Tai WM, Chung J, Tang PL, Koo YX, Hou X, Tay KW, Quek R, Tao M, Lim ST (2011). Central nervous system (CNS) relapse in diffuse large B cell lymphoma (DLBCL): pre- and post-rituximab. Ann Hematol.

[15] Batchelor TT, Grossman SA, Mikkelsen T, Ye X, Desideri S, Lesser GJ (2011). Rituximab monotherapy for patients with recurrent primary CNS lymphoma. Neurology.

[16] Shah GD, Yahalom J, Correa DD, Lai RK, Raizer JJ, Schiff D, LaRocca R, Grant B, DeAngelis LM, Abrey LE (2007). Combined immunochemotherapy with reduced whole-brain radiotherapy for newly diagnosed primary CNS lymphoma. J Clin Oncol.

[17] Siegal T (2014). Primary central nervous system lymphoma: current state of anti-CD20 therapy and appraisal of reported response criteria. J Clin Neurosci.

[18] Mocikova H, Pytlik R, Sykorova A, Janikova A, Prochazka V, Vokurka S, Berkova A, Belada D, Campr V, Buresova L, Trneny M (2016). Role of rituximab in treatment of patients with primary central nervous system lymphoma: a retrospective analysis of the Czech lymphoma study group registry. Leuk Lymphoma.

[19] Ly KI, Crew LL, Graham CA, Mrugala MM (2016). Primary central nervous system lymphoma treated with high-dose methotrexate and rituximab: A single-institution experience. Oncol Lett.

[20] Rubenstein JL, Combs D, Rosenberg J, Levy A, McDermott M, Damon L, Ignoffo R, Aldape K, Shen A, Lee D, Grillo-Lopez A, Shuman MA (2003). Rituximab therapy for CNS lymphomas: targeting the leptomeningeal compartment. Blood.

[21] Birnbaum T, Stadler EA, von Baumgarten L, Straube A (2012). Rituximab significantly improves complete response rate in patients with primary CNS lymphoma. J Neurooncol.

[22] Ott RJ, Brada M, Flower MA, Babich JW, Cherry SR, Deehan BJ (1991). Measurements of blood-brain barrier permeability in patients undergoing radiotherapy and chemotherapy for primary cerebral lymphoma. Eur J Cancer.

[23] Fritsch K, Kasenda B, Hader C, Nikkhah G, Prinz M, Haug V, Haug S, Ihorst G, Finke J, Illerhaus G (2011). Immunochemotherapy with rituximab, methotrexate, procarbazine, and lomustine for primary CNS lymphoma (PCNSL) in the elderly. Ann Oncol.

[24] Holdhoff M, Ambady P, Abdelaziz A, Sarai G, Bonekamp D, Blakeley J, Grossman SA, Ye X (2014). High-dose methotrexate with or without rituximab in newly diagnosed primary CNS lymphoma. Neurology.

[25] Rubenstein JL, Hsi ED, Johnson JL, Jung SH, Nakashima MO, Grant B, Cheson BD, Kaplan LD (2013). Intensive chemotherapy and immunotherapy in patients with newly diagnosed primary CNS lymphoma: CALGB 50202 (Alliance 50202). J Clin Oncol.

[26] Chamberlain MC, Johnston SK (2010). High-dose methotrexate and rituximab with deferred radiotherapy for newly diagnosed primary B-cell CNS lymphoma. Neuro Oncol.

[27] Ferreri AJ, Cwynarski K, Pulczynski E, Ponzoni M, Deckert M, Politi LS, Torri V, Fox CP, Rosee PL, Schorb E, Ambrosetti A, Roth A, Hemmaway C (2016). Chemoimmunotherapy with methotrexate, cytarabine, thiotepa, and rituximab (MATRix regimen) in patients with primary CNS lymphoma: results of the first randomisation of the International Extranodal Lymphoma Study Group-32 (IELSG32) phase 2 trial. Lancet Haematol.

[28] Madle M, Kramer I, Lehners N, Schwarzbich M, Wuchter P, Herfarth K, Egerer G, Ho AD, Witzens-Harig M (2015). The influence of rituximab, high-dose therapy followed by autologous stem cell transplantation, and age in patients with primary CNS lymphoma. Ann Hematol.

[29] Kansara R, Shenkier TN, Connors JM, Sehn LH, Savage KJ, Gerrie AS, Villa D (2015). Rituximab with high-dose methotrexate in primary central nervous system lymphoma. Am J Hematol.

[30] Liu J, Sun XF, Qian J, Bai XY, Zhu H, Cui QU, Li XY, Chen YD, Wang YM, Liu YB (2015). Immunochemotherapy for primary central nervous system lymphoma with rituximab, methotrexate, cytarabine and dexamethasone: Retrospective analysis of 18 cases. Mol Clin Oncol.

[31] Rubenstein JL, Fridlyand J, Abrey L, Shen A, Karch J, Wang E, Issa S, Damon L, Prados M, McDermott M, O'Brien J, Haqq C, Shuman M (2007). Phase I study of intraventricular administration of rituximab in patients with recurrent CNS and intraocular lymphoma. J Clin Oncol.

[32] Rubenstein JL, Li J, Chen L, Advani R, Drappatz J, Gerstner E, Batchelor T, Krouwer H, Hwang J, Auerback G, Kadoch C, Lowell C, Munster P (2013). Multicenter phase 1 trial of intraventricular immunochemotherapy in recurrent CNS lymphoma. Blood.

[33] Harris NL, Jaffe ES, Stein H, Banks PM, Chan JK, Cleary ML, Delsol G, De Wolf-Peeters C, Falini B, Gatter KC (1994). A revised European-American classification of lymphoid neoplasms: a proposal from the International Lymphoma Study Group. Blood.

[34] Trotti A, Colevas AD, Setser A, Rusch V, Jaques D, Budach V, Langer C, Murphy B, Cumberlin R, Coleman CN, Rubin P (2003). CTCAE v3.0: development of a comprehensive grading system for the adverse effects of cancer treatment. Semin Radiat Oncol.

